# Hyperglycemia-Induced Endothelial Dysfunction: From Classical Pathogenetic Mechanisms to Emerging Insights into ACE2 Protective Action

**DOI:** 10.3390/ijms27062660

**Published:** 2026-03-14

**Authors:** Giada Lodi, Domenico Sergi, Anna Dipinto, Francesca Bompan, Paola Secchiero, Rebecca Voltan, Arianna Romani

**Affiliations:** 1 Department of Translational Medicine and LTTA Centre, University of Ferrara, 44121 Ferrara, Italy; giada.lodi@unife.it (G.L.); paola.secchiero@unife.it (P.S.); 2 Department of Translational Medicine, University of Ferrara, 44121 Ferrara, Italy; domenico.sergi@unife.it; 3 Department of Environmental and Prevention Sciences and LTTA Centre, University of Ferrara, 44121 Ferrara, Italy; anna.dipinto@unife.it (A.D.); francesca.bompan@unife.it (F.B.); rebecca.voltan@unife.it (R.V.)

**Keywords:** CVD, hyperglycemia, diabetes, ACE2, endothelial dysfunction

## Abstract

Diabetes is a pivotal risk factor for cardiovascular disease as well as microvascular complications, including retinopathy and nephropathy. Chronic hyperglycemia is a key player in linking diabetes with endothelial dysfunction which, in turn, contributes to cardiovascular disease. Indeed, hyperglycemia acts as a trigger for endothelial dysfunction, promoting a shift in the endothelium from a protective, anti-inflammatory state to a dysfunctional, injury-prone phenotype. A hyperglycemic environment triggers several pathogenetic mechanisms, including alterations in bioenergetics, production of advanced glycation end products, oxidative stress and mitochondrial dysfunction, all contributing to endothelial dysfunction. The activation of these pathophysiological mechanisms by hyperglycemia culminates in reduced nitric oxide production, as well as the induction of oxidative stress and inflammation, all of which are pivotal in impairing endothelial homeostasis and promoting cellular damage. Besides these classical mechanisms, there is growing attention on novel pathogenetic factors linking diabetic hyperglycemia with endothelial dysfunction, such as the ACE2 protein. The latter is emergeing for its potential to counter hyperglycemia-induced cellular damage through its vasoprotective and anti-inflammatory actions, making it a promising therapeutic target for tackling endothelial dysfunction. This review provides an overview of classical as well as emerging mechanisms underpinning the deleterious effects of diabetic hyperglycemia on endothelial dysfunction. In turn, understanding the molecular interconnections between hyperglycemia and endothelial dysfunction is crucial for developing novel strategies to restore endothelial homeostasis and mitigate diabetic vascular complications.

## 1. Introduction

Diabetes mellitus (DM) is a metabolic disorder characterized by chronic hyperglycemia which develops as a consequence of an absolute insulin deficiency or insulin resistance. While the first represents the pathogenetic basis of type 1 (T1DM), the latter underpins the onset of type 2 diabetes mellitus (T2DM). The prevalence of diabetes has reached epidemic proportions in developed and, increasingly, in developing countries [1]. Most importantly, the diabetes epidemic is still on the rise with 1.31 billion people expected to suffer from diabetes by 2050 [2]. This diabetes epidemic is driven by the alarming rise in the cases of T2DM which represents 90% of all cases of diabetes and is a direct consequence of the upsurge in the prevalence of obesity. Nevertheless, the incidence of T1DM is also increasing in parallel with T2DM [3]. The unsustainable health and economic burden of diabetes strives from its global epidemic proportions as well as the life-threatening consequences of these metabolic disorders. Indeed, diabetes must not be regarded merely as a disruption of glucose, lipid, and protein metabolism; instead, it represents a pivotal risk factor for a plethora of comorbidities, including microvascular complications, cardiovascular disease (CVD), several types of cancer, kidney and neurodegenerative diseases [4,5,6,7,8,9].

CVD is a prominent comorbidity of diabetes, given the fact that it represents the primary cause of death worldwide [10] and the leading cause of mortality among individuals living with T1DM and T2DM [11,12]. The defective metabolic control typical of diabetes and particularly related to glucose and lipid metabolism is a key driver of its micro- and macrovascular complications which, in turn, underlay the development of CVD, nephropathy and retinopathy. In particular, hyperglycemia, along with diabetic dyslipidemia, negatively impacts the micro- and macrovasculature, thereby contributing to the pathogenesis of atherosclerosis and CVD [13,14].

Microvascular endothelial cell dysfunction mainly compromises retinal and glomerular endothelial cells of diabetic patients. This causes glomerular and retinal endothelial barrier breakdown, compromising their function [15]. Microvascular disorders have been reported to occur before diabetes diagnosis, and they might increase the risk for CVD. Even if no clear data explaining this association are available, clinical evidence supporting this relationship is available and has been revised by Yu and colleagues [16].

Hyperglycemia is able to trigger a wide array of mechanisms, ranging from inflammation to oxidative stress and defective mitochondrial dynamics [17], which culminate with the onset of endothelial dysfunction.

In agreement with this, endothelial dysfunction can discriminate individuals at higher vascular risk, constitute an early indicator and a key pathogenetic trigger for the onset of atherosclerosis or microvascular dysfunction. Thus, targeting the mechanisms by which hyperglycemia induces endothelial dysfunction represents a promising approach for preventing CVD. In light of this, this review aims to provide an updated overview of classical and emerging molecular mechanisms underlying the effects of hyperglycemia on endothelial dysfunction.

## 2. Endothelial Dysfunction and Vascular Disease

The endothelium is a semipermeable barrier made up of a single-cell monolayer that lines the entire vasculature and is responsible for the exchange of nutrients, metabolites, and fluids between the blood and tissues [18]. However, this definition of endothelium is all but comprehensive. Indeed, the endothelium is also involved in preventing clot formation and acts as a source of bioactive molecules, including vasoconstrictors, vasodilators, and regulators of thrombosis and inflammation [19]. These mediators, in turn, are responsible for regulating vessel tone, platelet activation, vascular smooth muscle cell proliferation and leukocyte adhesion [20]. A pivotal driver of endothelial function is nitric oxide (NO), which is synthesized by endothelial nitric oxide synthase (eNOS). NO exerts vasodilatory, anti-thrombotic, and anti-inflammatory effects, which are all pivotal for maintaining vascular homeostasis [21]. Maintaining endothelial health is crucial in sustaining vascular homeostasis. It translates that endothelial dysfunction, defined as any pathophysiological changes that negatively impact the vasoprotective function of the endothelium, hampers vascular health, paving the way for the development of vascular complications of diabetes, including CVD [17,20,22,23]. Defective endothelial function is not intended as a single pathological state but rather a spectrum of phenotypes associated with heterogeneous pathophysiological alterations, including altered vascular tone, permeability, inflammation, and de-differentiation, leading to the loss of endothelial homeostatic functions [18,24]. The activation of these mechanisms, in turn, represents the basis for the development of atherosclerosis, driven by impaired vasoregulation and unresolved chronic inflammation [19]. Indeed, the endothelium not only has a pivotal role in atherosclerosis initiation, but also in plaque progression. Hyperglycemia, along with other insults reviewed elsewhere [25,26], represents a crucial player in triggering the pathophysiological mechanisms underpinning endothelial dysfunction and endothelial cell activation.

**Figure 1:**
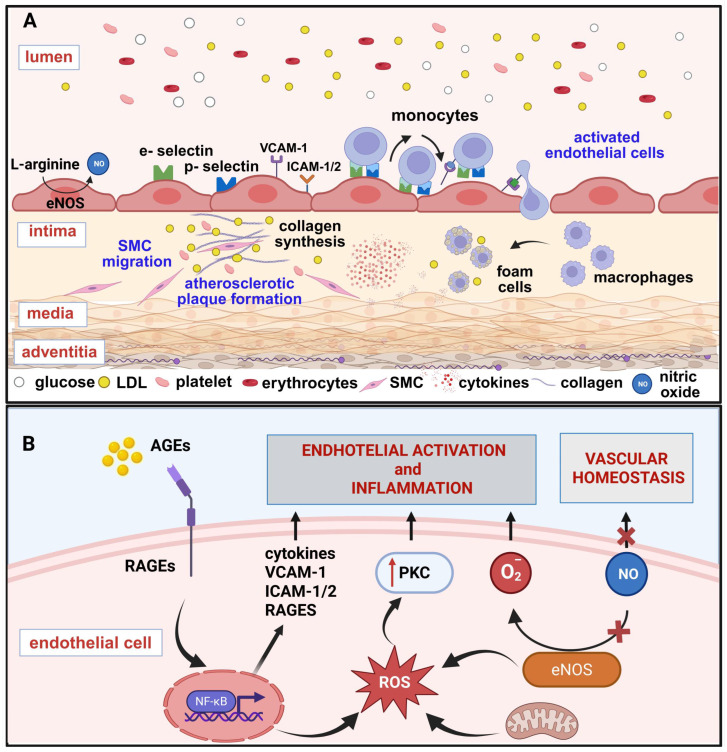
Mechanisms of endothelial activation and dysfunction under hyperglycemic conditions. (**A**) Hyperglycemia induces endothelial activation in the blood vessel lumen, where elevated glucose and LDL levels cause a crucial reduction in bioavailable in NO produced by eNOS, and the expression of adhesion molecules on endothelial cells, including E-selectin, P-selectin, VCAM-1, and ICAM-1/2. These processes lead to increased monocyte adhesion and transmigration into the subendothelial intima space. These cells accumulate cholesterol to transform into foam cells, setting the scene for the development of atherosclerotic plaques. (**B**) Schematic representation of intracellular mechanisms contributing to endothelial dysfunction. Hyperglycemia induces eNOS uncoupling, thereby reducing NO production by increasing superoxide generation. Mitochondrial overproduction of ROS acts as a signaling mechanism that enhances (↑) protein kinase C (PKC) activation that causes endothelial activation and inflammation. AGEs bind to their receptor, RAGE, inducing a pro-inflammatory state through the activation of nuclear factor kappa-light-chain-enhancer of activated B cells (NFκB) signaling. This cascade drives the expression of pro-inflammatory cytokines, adhesion molecules (VCAM-1, ICAM-1/2), and a positive feedback loop that increases RAGE expression. Created in BioRender. Lodi, G. (2026) https://BioRender.com/65untio (accessed on 11 March 2026).

Endothelial cells, upon activation, express a variety of molecules like ICAM, VCAM, E- and P-selectin, which promote the attraction and adhesion of neutrophils and monocytes that penetrate the arterial wall [27]. Subsequently, monocytes differentiate into macrophages within the arterial wall and start accumulating lipids, particularly by scavenging oxidized low-density lipoproteins (oxLDL), to become foam cells, which in turn, initiate the formation of atherosclerotic plaque. Excessive accumulation of foam cells forms a necrotic core in atherosclerotic plaque, increasing the risk for vascular rupture [28] (Figure 1A). At the same time, the infiltration of immune cells into the arterial vessel also fuels vascular inflammation through direct secretion of TNF-α and IL-1β and stimulation of endothelial cells to release pro-inflammatory mediators, such as IL-6 [27,29,30].

## 3. Hyperglycemia and Endothelial Dysfunction

High glucose levels are considered as a pivotal driver of endothelial dysfunction [17,31], with this paradigm supported by cell and animal studies as well as clinical evidence.

In diabetic individuals, hyperglycemia is associated with abnormal endothelial morphology, as indicated by increased corneal thickness, enlarged endothelial cells, and decreased cell density lining the endothelium, as observed by non-contrast specular microscopy [32].

Furthermore, Chen and colleagues demonstrated that extended exposure (72 h) of corneal endothelial cells to 30 mM glucose significantly reduced adhesion and barrier proteins (N-cadherin/zonulin-1), water channels (aquaporin-1), and Na^+^/K^+^ ATPase protein (ATP1a1). This was accompanied by endothelial dysfunction and impaired mitophagy, leading to the accumulation of damaged mitochondria. In support of this evidence, exogenous application of the mitophagy agonist carbonyl cyanide m-chlorophenyl hydrazine (CCCP) protected the corneal endothelium from hyperglycemia-induced damage [32].

In Human Umbilical Vein Endothelial Cells (HUVECs), a 24 h high-glucose treatment markedly worsened the effects of an inflammatory state, such as that induced by SARS-CoV-2 infection [33]. Cells treated with spike protein under hyperglycemia-mimicking conditions showed increased ROS production due to upregulation of NADPH oxidase and a simultaneous decrease in angiotensin-converting enzyme 2 (ACE2) protein levels, compared to spike protein-treated cells in standard culture conditions. Junction proteins were also reported to be downregulated in response to high glucose levels, impairing endothelial polarity and barrier integrity in the same cells [33].

However, high glucose exposure of HUVECs in the absence of additional inflammatory stimuli was reported to be insufficient to modulate ROS production or the expression of junction proteins such as zonulin-1 and occludin [33].

Furthermore, high glucose levels have been reported to reduce the number and function of endothelial progenitor cells maintained for 7 days with 30 mM glucose and increase endothelial autophagy, restraining vascular repair capacity and accelerating vascular injury [34]. In agreement, Niu and colleagues observed autophagy activation both in vivo and in vitro [35]. In diabetic mice, retinal endothelial cells exposed to high glucose displayed impaired tube formation, increased apoptosis mediated by caspases (−9, −8, −3), and enhanced autophagy, evidenced by elevated autophagosome formation and LC3-II expression.

In animal models, hyperglycemia worsened endothelial dysfunction in hyperhomocysteinemic mice by activating µ-calpain and inhibiting endothelial eNOS, thereby reducing NO bioavailability [36]. Nevertheless, in rat ocular endothelial cells exposed to 24 mM glucose for 2 h, Qambari and colleagues reported a significant vasodilatory response, which was further exacerbated in endothelial cells from diabetic rats [37]. These results support the existence of compensatory mechanisms that promote vasodilation despite decreased NO availability caused by acute hyperglycemia. These mechanisms effectively counteract the detrimental effects of high glucose levels in the early stages of diabetes but eventually become overwhelmed as hyperglycemia becomes chronic [37].

Similarly, an antioxidant compensatory response to short-term hyperglycemia has also been described in bovine retinal endothelial cells. In these cells, the induction of manganese superoxide dismutase (MnSOD) or uncoupling proteins (UCPs) mitigated the injury induced by moderate hyperglycemia. However, this compensatory response failed as glucose levels increased, leading to structural damage and cell loss primarily driven by hyperactive mitochondria [38].

## 4. Molecular Mechanisms of Hyperglycemia-Induced Cellular Damage

Chronic hyperglycemia leads to the development of endothelial damage and dysfunction via a wide array of mechanisms, which include the activation of the PKC, the polyol and hexosamine pathways and by promoting the non-enzymatic formation of advanced glycation end products (AGEs) [39]. Furthermore, the detrimental effects of glucotoxicity on the endothelium are also mediated by increased ROS formation and the resulting oxidative stress [40] (Figure 1B).

### 4.1. The Activation of the Polyol and the Hexosamine Pathways

Increased glucose intracellular availability, secondary to hyperglycemia, is pivotal in promoting glucotoxicity. Indeed, as glucose rises intracellularly, its flux towards the polyol and the hexosamine pathways raises with a parallel decrease in the amount of glucose fueling the pentose phosphate pathway and glycolysis [40]. In this regard, as glucose is funneled towards the polyol pathway, it is initially converted to sorbitol by aldose reductase. Sorbitol is then used as a substrate for sorbitol dehydrogenase leading to the synthesis of fructose [41,42]. The activation of this pathway not only leads to an intracellular increase in sorbitol and fructose but also depletes NAD^+^ as well as NADPH. The increases in sorbitol and fructose contribute to cellular damage through two separate mechanisms. While sorbitol fosters osmotic stress, increased intracellular fructose levels are converted to the highly reactive α-oxo-aldehyde 3-deoxyglucosone, thereby promoting AGE production [43]. The depletion of NAD^+^, as a consequence of the activation of the polyol pathway, is responsible for the inhibition of the enzyme glyceraldehyde 3-phosphate dehydrogenase, which, in turn, is a key node of the glycolytic pathway [7]. In addition, the activation of the polyol pathway disrupts the NAD^+^/NADH ratio with an increase in NADH flux through the electron transport chain promoting the production of superoxide, hydrogen peroxide and hydroxyl radicals [44,45]. The contribution of the polyol pathway to oxidative stress is further exacerbated by the depletion of NADPH which is pivotal for the regeneration of reduced glutathione, thereby causing an imbalance between pro-oxidant species and antioxidant defense systems [40,46]. Regarding the hexosamine pathway, it is a secondary route of glucose metabolism that is stimulated by hyperglycemia. Indeed, in response to hyperglycemia, excess fructose-6-phosphate, arising from impaired glucose metabolism, is converted to UDP-N-acetylglucosamine (UDP-GlcNAc) [42]. UDP-GlcNAc, in turn, induces posttranslational modifications that compromise cytoplasmic and nuclear protein function thereby impairing cellular homeostasis [47]. A key protein whose posttranslational modification by UDP-N-acetylglucosamine contributes to endothelial dysfunction is represented by eNOS. In this regard, the O-GlcNAcylation of eNOS at the AKT phosphorylation target site inhibits its function [48]. This translates into a reduction in NO production, a crucial aspect of the pathogenesis of endothelial dysfunction [49].

### 4.2. Hyperglycemia-Induced Protein Kinase C Activation

The activation of PKC, particularly the isoform PKC-β [45], is a further mechanism mediating the detrimental effects of hyperglycemia on endothelial function. The activation of PKC as a consequence of hyperglycemia is driven by the intracellular buildup of ROS, AGEs and diacylglycerol [40]. In particular, the accumulation of diacylglycerol is a direct consequence of the intracellular increase in glyceraldehyde 3-phosphate. The latter increases intracellularly due to NAD+ depletion, resulting in inhibition of glyceraldehyde 3-phosphate dehydrogenase. PKC overactivation contributes to endothelial dysfunction and atherosclerotic plaque formation by promoting a pro-inflammatory and pro-fibrotic environment in the vasculature, impairing vascular insulin signaling, inducing adhesion molecule expression, and downregulating eNOS [49,50]. Additionally, PKC promotes activation of NADPH oxidase, thereby fueling oxidative stress and lowering nitric oxide bioavailability with a consequent impairment in endothelium-dependent vasodilation [49,51]. In agreement with the detrimental role of PCK, its inhibition has been shown to mitigate endothelial dysfunction triggered by hyperglycemia [52].

### 4.3. The Formation of Advanced Glycation End Products

**Figure 2:**
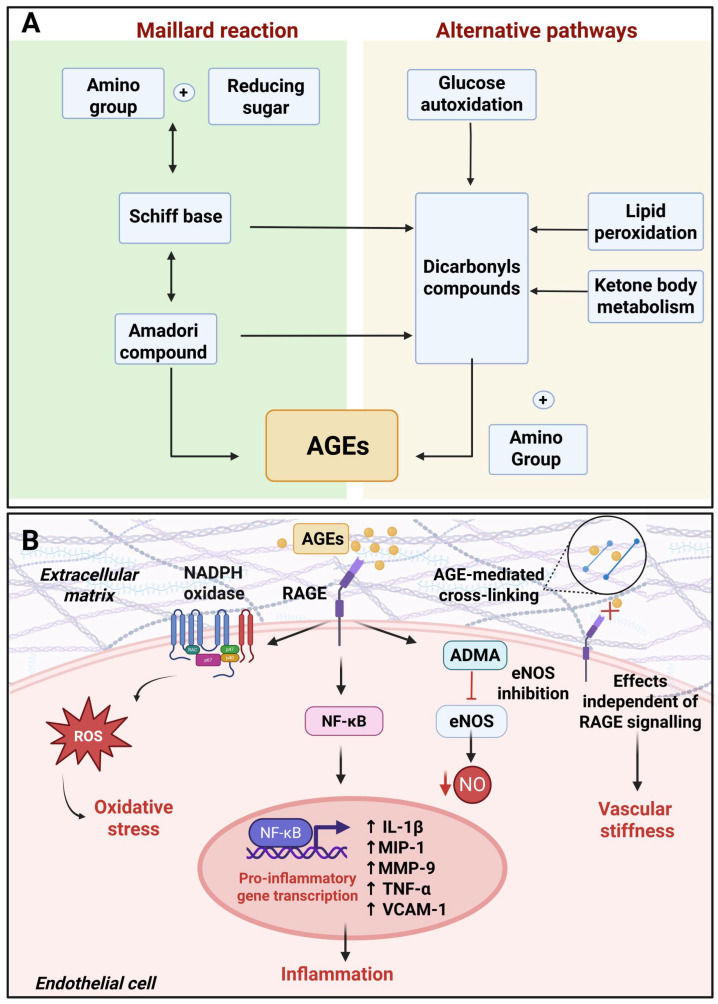
AGE formation and signaling cascade in hyperglycemic conditions. (**A**) Left: the Maillard reaction involves the formation of a Schiff base from the reaction between reducing sugars with amino groups of proteins, followed by Amadori rearrangement to generate Amadori products. Right: alternative pathways, including glucose autoxidation, ketone body metabolism, or lipid peroxidation, produce reactive dicarbonyl intermediates that further react to form irreversible products, AGEs. (**B**) AGEs accumulate in the extracellular matrix and form cross-links with structural proteins such as collagen, laminin, and elastin, increasing vascular stiffness. In addition to direct protein glycation, AGEs bind to their receptor (RAGE), activating intracellular signaling pathways that enhance oxidative stress and inflammation. AGE–RAGE interaction promotes NADPH oxidase activation and ROS production, inhibits eNOS, reducing (↓) NO production, and triggers activation of NFκB, which translocates to the nucleus and induces the transcription (↑) of pro-inflammatory cytokines, growth factors, and adhesion molecules. Created in BioRender. Lodi, G. (2026) https://BioRender.com/ayzi3mx (accessed on 11 March 2026).

Hyperglycemia is a key driver of endogenous AGE production, which represents an additional mechanism by which glucotoxicity contributes to endothelial dysfunction. The non-enzymatic formation of AGEs is the result of the interaction between reducing sugars, such as glucose and fructose, and the free amino groups of lipid, protein and nucleic acids. This reaction generates unstable Schiff bases, which undergo further rearrangements to produce Amadori products that accumulate on proteins, leading to the formation of AGEs [53,54]. In this regard, non-enzymatic protein glycation induces functional modifications that disrupt protein function (Figure 2A). These modifications also affect the extracellular matrix with AGE cross-linking with collagen, laminin, and elastin, leading to an increase in vascular stiffness [55]. In support of this, individuals with T2DM are characterized by increased vascular stiffness [56]. However, the detrimental effects of AGEs span beyond protein glycation. Indeed, AGEs can bind to their cognate receptor, the receptor for AGEs (RAGE), triggering the activation of intracellular pathways that exacerbate oxidative stress and inflammation [53]. The AGE–RAGE interaction promotes the activation of the nuclear factor kappa-light-chain-enhancer of activated B cell (NFκB) pathway, which induces the transcription of growth factors and adhesion molecules. Furthermore, AGEs activate extracellular signal-regulated kinase (ERK) 1/2, mitogen-activated protein kinase (MAPK) and Janus kinase (JAK) which, in concert with NFκB signaling, promote the expression of interleukin-1β, macrophage inflammatory protein-1, matrix-metalloproteinase 9, TNF-α and VCAM-1 responsible for promoting leucocyte adhesion and vascular inflammation [57]. The activation of MAPK, in concert with the upregulation of platelet-derived growth factor and transforming growth factor-beta (TGF-β), triggered by AGEs, stimulates vascular smooth muscle cells proliferation and migration [58,59]. These mechanisms, in turn, contribute to arterial remodeling and neointima formation [60]. Moreover, AGEs contribute to impaired angiogenesis by inhibiting endothelial progenitor cell migration and tube formation [61], potentially by inhibiting galectin-3 interaction with its receptor integrin α5β1 [62]. Remarkably, the impairment of galectin-3 binding to integrin α5β1 p also contributes to impaired diabetic wound healing [62]. AGEs also promote endothelial-to-mesenchymal transition as demonstrated by the upregulation of fibroblast-specific protein-1, α-smooth muscle antibody and collagen I and a downregulation of cluster of differentiation 31 in AGE-treated HUVECs [63]. In particular, the endothelial-to-mesenchymal transition triggered by AGEs relies on the upregulation of TGF-β and the downregulation of silent mating type information regulation 2 homolog 1 (Sirt 1) [63] as well as via protein kinase B signaling cascades [64]. Thus, endothelial-to-mesenchymal transition, by impairing endothelial cell specialized phenotype, represents an additional mechanism underpinning AGE-induced endothelial dysfunction. Apart from sustaining chronic inflammation-induced vascular damage, the activation of RAGE also culminates with NADPH oxidase which reiterates oxidative stress [40]. Moreover, upon binding on RAGE, AGEs reduce eNOS activity by inducing its inhibitor, asymmetric dimethylarginine [65]. Furthermore, AGEs disrupt the tight junctions between endothelial cells and promote the degradation of endothelial glycocalyx, thereby increasing endothelial permeability [42,66]. While diabetic hyperglycemia is pivotal in promoting AGE production, it is not the only factor driving the generation of these molecules. As such, while AGEs activated inflammatory responses, chronic inflammation itself promotes AGE generation, thereby generating a self-sustaining vicious cycle (Figure 2B).

### 4.4. Oxidative Stress and Mitochondrial Dysfunction

Hyperglycemia is pivotal in fostering ROS production, which represents the major contributor to endothelial dysfunction [67]. Indeed, increased glucose catabolism, secondary to hyperglycemia, results in a rise in the electron donors NADH and FADH_2_ which are funneled to the mitochondrial electron transport chain. The enhanced flux of electrons through the electron transport chain leads to an increase in mitochondrial membrane potential which, once reaches a critical threshold, impairs electron transport at complex III [42,68]. This scenario favors electron leakage which are transferred, one at the time, to molecular oxygen, thereby generating superoxide [42]. In terms of the mechanisms by which oxidative stress contributes to endothelial dysfunction, these include the activation of pro-inflammatory responses, the decrease in NO bioavailability as well as ROS-induced mitochondrial dysfunction [69]. Oxidative stress can directly lower NO bioavailability. In this regard, NO is converted to ONOO^−^ by reacting with superoxide radical [70]. Additionally, oxidative stress can downregulate eNOS expression, promote its uncoupling and lower the substrates this enzyme requires for the synthesis of NO, such as L-arginine [71]. Oxidative stress can also exacerbate vascular inflammation by activating the NFκB signaling pathway [72] and promoting the expression of IL-1β, IL-6, TNF-α as well as adhesion molecules, including VCAM-1 and ICAM-1 [69]. Additionally, excessive ROS production also hampers mitochondrial function which, in turn, contributes to endothelial dysfunction [73]. Indeed, mitochondria are not only a pivotal source of ROS, but ROS themselves can disrupt mitochondrial health. As such, ROS can hamper mitochondrial calcium homeostasis [74], damage the proteins of the mitochondria electron transport chain as well as mitochondrial DNA and disrupt energy production [75,76]. Thus, impaired mitochondrial health represents an additional mediator linking excessive ROS production, oxidative stress, and endothelial dysfunction.

Hyperglycemia and ROS-induced mitochondrial fission have been shown to play a crucial role in fostering mitochondrial dysfunction [69,77,78]. Mitochondria undergo continuous cycles of fusion and fission via a process termed mitochondrial dynamics. These cycles of fusion and fission are pivotal to allow the cells to adapt to the metabolic fuel availability, cellular energy demand, to respond to different stressors and they are required for mitochondrial quality control [79]. Despite both fusion and fission being required to maintain mitochondrial health and cellular homeostasis, an imbalance in mitochondrial dynamics impairs cellular metabolic health. Indeed, while mitochondrial fission under physiological conditions represents a beneficial process allowing the removal of damaged mitochondrial components through mitophagy, excessive fission can be detrimental. Indeed, an increase in fission leads to impaired energy production and constitutes an early event preceding ROS overproduction as well as apoptosis in response to hyperglycemia [80]. Most importantly, mitochondrial fragmentation is instrumental for hyperglycemia-induced excessive ROS production [80], highlighting impaired mitochondrial dynamics as a key pathophysiological mechanism linking high glucose levels and endothelial dysfunction. Moreover, an imbalance in mitochondrial dynamics, with a shift towards fission, contributes to endothelial inflammation, impairs endothelial-dependent relaxation and hampers angiogenesis as well as wound healing [77,81]. Thus, defective mitochondrial dynamics with an increase in fission or decrease in fusion underpins an increase in mitochondrial fragmentation and compromises endothelial physiological function [82]. In terms of the mechanisms underlying the ability of hyperglycemia to promote mitochondrial fission, these relay on an increase in ROS production as well as the modulation of the activity of dynamin-related protein 1 (DRP1), a critical actor for promoting mitochondrial fragmentation [76]. Indeed, under hyperglycemic conditions, DRP1 is phosphorylated on serine600 by Rho-associated coiled coil-containing protein kinase 1 (ROCK1) resulting in its translocation to the mitochondria where it orchestrates the fission of these organelles [83]. However, other evidence indicates that hyperglycemia promoted the activation of DRP1 by promoting ROCK1-mediated phosphorylation on serine616 [84]. Additionally, ROCK1 was identified as a downstream target of Forkhead box protein O1 (FOXO1) whose inhibition was sufficient to prevent hyperglycemia-induced mitochondrial fission [84].

Even though mitophagy is crucial for mitochondrial quality control, its deregulation contributes to endothelial dysfunction. Hyperglycemia is able to induce mitophagy [85] which, in turn, represents a further mitochondrial mechanism by which hyperglycemia contributes to endothelial dysfunction. Short-term high glucose treatment has been reported to induce the Parkin pathway, while long-term hyperglycemia inhibits the Parkin pathway and activates sintaxin 17-mediated mitophagy, promoting endothelial dysfunction and vascular injury [85]. The relevance of deregulated mitophagy in the context of endothelial dysfunction has been confirmed by silencing sintaxin-17 in endothelial cells exposed to long-term hyperglycemia. Indeed, the inhibition of mitophagy by sintaxin-17 reduced endothelial dysfunction as indicated by a decrease in ROS, apoptosis and a parallel increase in eNOS phosphorylation [85].

## 5. Renin-Angiotensin System Regulation in Hyperglycemia

The renin-angiotensin system (RAS) is essential for the regulation of blood pressure and body fluid balance. Circulating renin, produced by the juxtaglomerular cells in the kidneys, cleaves liver-derived angiotensinogen into angiotensin I (AngI) which is then hydrolyzed by endothelial ACE to form angiotensin II (Ang II). Ang II binds to the angiotensin II type 1 receptor (AT1R), stimulating aldosterone production and vasoconstriction. Alternatively, Ang II can bind the angiotensin II type 2 receptor (AT2R) to counteract vasoconstriction [86].

ACE2 receptor, a homolog of the classical ACE, can bind Ang II, converting it into Ang-(1-7), a peptide that via Mas1 receptor counteracts the inhibitory effects of Ang II on insulin release by modulating cAMP levels and eNOS activity. It dampens inflammation and promotes antioxidant as well as vasodilatory effects [86].

In diabetes, systemic RAS has been reported to be suppressed, whereas local RAS (e.g., in the kidney, pancreas, and adipose tissue) has been shown to be activated. Its regulation in response to increasing glucose concentrations depends on cell type and angiotensin receptor levels [87,88]. In diabetic rat kidneys, increased renin gene and protein expression were associated with increased Ang II production and downregulation of AT2R, supporting the detrimental effects of Ang II. In endothelium, Ang II/AT1R stimulates free radical production through NADPH oxidase, promoting insulin resistance and ultimately exacerbating macrovascular complications of diabetes [88]. Hyperglycemia promotes ACE2 glycation that reduces its enzymatic activity and stability. In vitro data showed increased non-enzymatic ACE2 glycation in cardiomyocytes exposed to 120 mM glucose for 12 days [89]. In agreement with this notion, increased levels of glycated ACE2 and reduced expression of Ang-(1-9), Ang-(1-7) and MasR were reported in cardiomyocytes from T2DM patients compared with those derived from healthy subjects [89,90].

**Figure 3:**
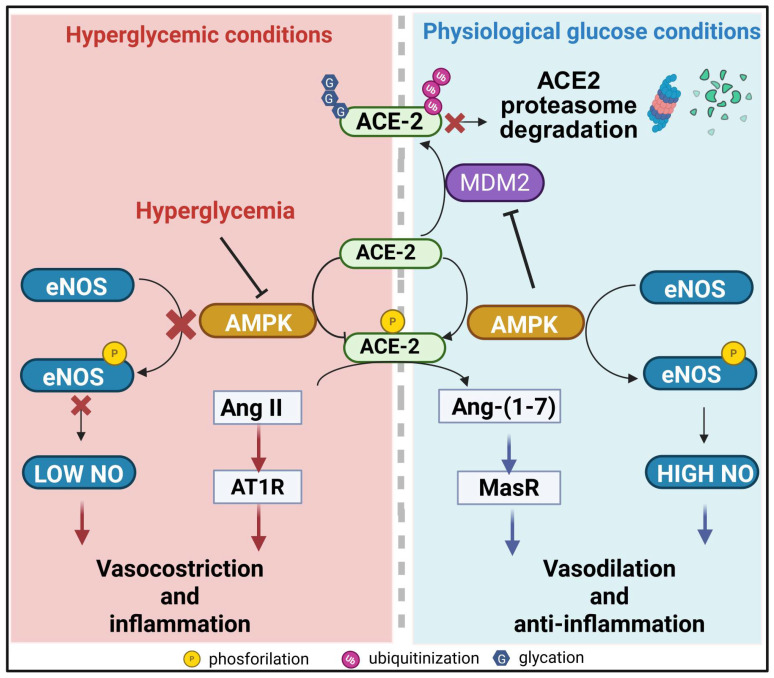
ACE2 regulation under normoglycemic and hyperglycemic conditions. Under physiological conditions (right panel) AMPK-mediated phosphorylation enhances ACE2 activity and inhibits MDM2-mediated ubiquitination, promoting the conversion of Ang II to Ang-(1-7), which exerts vasodilatory and anti-inflammatory actions mediated by the MAS receptor (MasR). AMPK simultaneously activates eNOS, NO production and it supports endothelial homeostasis. Under hyperglycemic conditions (left panel), reduced AMPK activity, followed by reduced eNOS phosphorylation, leads to less nitric oxide availability. In parallel, impaired AMPK signaling fails to phosphorylate/activate ACE2. This promotes Ang II accumulation, which exerts vasoconstrictive and pro-inflammatory actions, ultimately contributing to endothelial dysfunction. Created in BioRender. Lodi, G. (2026) https://BioRender.com/emptppy (accessed on 11 March 2026).

Post-transcriptional modifications of ACE2, such as phosphorylation or glycation, interfere with its enzymatic or binding activity, ultimately impairing its vasoprotective role within the RAS pathway [89,91]. In pulmonary endothelial cells, impaired AMP-activated protein kinase (AMPK)-dependent phosphorylation of ACE2 promotes ACE2 degradation through Murine Double Minute 2 (MDM2), leading to pulmonary hypertension, which is linked to a significantly higher risk of right-heart failure and overall cardiovascular disease burden (Figure 3) [92,93]. Reduction in ACE2 bioavailability can also be mediated by due disintegrin and metalloprotease 17 (ADAM17)-induced shedding. The “shaddase” protease activity of ADAM17 removes the ectodomain of the transmembrane protein ACE2, resulting in the release of soluble ACE2. In diabetic mouse models, both T1DM and T2DM, higher levels of ADAM17 correlate with increased production of soluble ACE2, which is then excreted in urine [94]. This mechanism reduces ACE2 activity as a transmembrane protein, promoting plaque formation and a pro-atherosclerotic environment [95].

## 6. The Protective Role of ACE2 in Endothelial Dysfunction

The protective activity of ACE2 on endothelial cells is mainly driven by its ability to counteract the harmful effects of Ang II, as shown in both in vitro and in vivo studies.

In this regard, ACE2 KO mice were characterized by an impaired vasodilation of cerebral arteries which worsened the risk of stroke [96]. In adult mice, ACE2 depletion was associated with endothelial dysfunction that was exacerbated by oxidative stress during aging [96].

ACE2 expression was reported to be upregulated in pancreatic islet cells of diabetic mice, where it counteracts the activation of the pancreatic RAS. Thus, ACE2 may dampen the deleterious effects of RAS overactivation on β-cell and microvascular endothelial cell function [97,98].

In diabetic patients, plasma ACE2 levels follow a biphasic fluctuation that correlates with disease progression [99].

In pre-diabetic individuals, increased levels of ACE2 have been reported as a compensatory response to RAS activation aimed at balancing Ang II activity and preventing local and systemic vascular complications [97]. Although in the late stages of diabetes, the balance between Ang II and ACE2/Ang-(1-7) was compromised, possibly by the activation of pro-inflammatory responses [99].

Based on these premises, rebalancing RAS by targeting ACE2 pathways has been investigated by several studies in order to contrast cardiovascular complications, including endothelial dysfunction.

Exogenously administered Ang-(1-7) relieves cytotoxicity, apoptosis, and inflammation and counters the reduction in eNOS in HUVECs maintained in a high-glucose environment [91]. Ang-(1-7) exerts these effects by inhibiting activation of the JAK/STAT3 pathway, known to be associated with the progression of cardiovascular complications [91,100].

In the retinal endothelium, ACE inhibitors have been shown to restore the balance between classical (ACE1/Ang II) and vasoprotective (ACE2/MasR) pathways, lowering the upregulation of both ACE1 and Ang II reported in ocular tissues. Overexpression of ACE2 through adeno-associated virus inhibited the formation of acellular capillaries and limited the release of proinflammatory mediators from the retina, preventing or reversing retinopathy in diabetic mice [101,102].

ACE2 has also been reported to act as a protective factor in the kidneys, where its counterregulatory effect on Ang II reduces the risk of diabetic nephropathy [103]. In the kidney, endothelial cells release pivotal factors involved in vascular homeostasis, and their dysfunction is associated with the onset and progression of chronic kidney disease (CDK), a multisystemic disorder that increases the risk of CVD [104].

## 7. RAS and ACE2 as Therapeutic Targets for Endothelial Dysfunction

ACE2 functions and its potential as a therapeutic target have attracted a great deal of attention since the COVID-19 pandemic. Nevertheless, its pathophysiological relevance spans beyond its role as an entry receptor for SARS-CoV2. Indeed, ACE2 constitutes a promising target for preventing vascular complications related to RAS dysregulation [86,105]. Nevertheless, to date, no specific molecules targeting ACE2 have been approved, even though several trials are still ongoing.

Pharmacological RAS blockade with angiotensin-converting enzyme inhibitors (ACEis) or angiotensin receptor blockers (ARBs) has demonstrated cardioprotective and renoprotective effects, by counteracting Ang II activity [106].

ACEis and ARBs have different sites of action; ACEis are competitive inhibitors of ACE, which inhibit the conversion of AngI to Ang II, while the ARBs are highly selective for the AT_1_ receptor and block the deleterious effects of Ang II [107,108].

The two agents do not directly impact ACE2 expression but indirectly increase ACE2 activity by rebalancing the ACE1/ACE2 ratio. This shifts the RAS balance toward increased production of Ang-(1-7), which, as already described, exerts vasodilatory, anti-inflammatory, and anti-fibrotic effects [109]. A meta-analysis of randomized controlled studies, which involved almost 21,871 hypertensive patients with T2DM, demonstrated that ACEi/ARBs led to a 10% decrease in cardiovascular events and 17% reduction in cardiovascular mortality when compared with control or other antihypertensive therapies [110].

To date, two phase IV studies investigating the effects of two different ARBs on circulating ACE, ACE2, Ang II, and Ang-(1-7) levels, as well as vascular function parameters in hypertensive patients with T2DM, are published on ClinicalTrials.gov and they are still ongoing. These trials compare two different ARBs, olmesartan and fimasartan, with the conventional antihypertensive agent amlodipine.

The clinical trial investigating olmesartan action (NCT05189015) has completed the enrolment of 80 participants, but no results have been published yet, while the clinical trial investigating the fimasartan activity is still recruiting patients (NCT05173025).

In the European Union there is one ongoing registered trial aimed at investigating the effect of empagliflozin on RAS in T2DM patients with chronic kidney disease (EudraCT Number 2016-002935-14) (www.clinicaltrialsregister.eu). Empagliflozin inhibits the sodium-glucose co-transporter-2 (SGLT-2), reducing renal reabsorption of glucose and increasing its urinary excretion. Empagliflozin use in diabetes has already been approved by the Food and Drug Administration (FDA); however, new therapeutic indications are under investigation.

Emerging therapies targeting the mineralocorticoid receptor (MR) may offer alternative strategies for reducing cardiovascular and renal events in patients with T2DM [111]. A clinical study completed in 2014 evaluated whether mineralocorticoid receptor antagonists exert beneficial cardiovascular effects by reducing vascular injury and improving vascular function. In this randomized, double-blind study, 64 participants with T2DM were treated with Spironolactone or Hydrochlorothiazide, reporting comparable reductions in systolic blood pressure, while only spironolactone improved coronary microvascular function [112].

This study suggests mineralocorticoid agonists might confer additional cardiovascular protection compared to ACE1 inhibitors in patients with T2DM.

Spironolactone presents antiandrogen and antiprogesterone side effects that might be overcome with eplerenone. In this regard, in the Australian–New Zealand clinical trials registry, one trial aims to investigate “aldosterone receptor” antagonist eplerenone in combination with standard treatment for T1DM in order to delay or prevent diabetes complication progression (ACTRN12613001355763) (www.anzctr.org.au). The study is not recruiting yet.

Apparently, no studies on the RAS pathway factors in diabetes are listed in the Chinese Clinical Trial registry (www.chictr.org.cn).

A novel approach to stabilize ACE2 protein involves using MDM2 inhibitors, which hamper MDM2-dependent ubiquitination of ACE2 [93].

MDM2 inhibitors are primarily used in cancer; recently, they have also been investigated for chronic diseases such as cardiovascular disease, diabetes, inflammatory and autoimmune disease [113]. Preclinical data reported both anti-inflammatory and anti-angiogenic effects, limiting new vessel formation by inhibiting endothelial cell proliferation, migration, and tube formation in vitro and in vivo [114,115]. This activity might prevent the formation of impaired new vessels originating from dysfunctional endothelial cells. Based on these premises, MDM2 inhibitors may harbor promising activity in regulating endothelial function and warrant further investigation.

## 8. Conclusions

Hyperglycemia significantly contributes to endothelial dysfunction via interconnected pathways underpinning atherosclerosis and diabetic vascular complications. The endothelium represents a promising therapeutic target for preventing and mitigating diabetes-related vascular disease. Despite significant advances in understanding the molecular basis of hyperglycemia-induced endothelial dysfunction from in vitro studies and animal models, therapeutic strategies to tackle endothelial dysfunction remain elusive. In this context, ACE2 represents a promising molecular target to counter the detrimental effects of hyperglycemia on endothelial dysfunction with potential therapeutic repercussions on diabetic vascular complications. Nevertheless, despite putative beneficial effects of ACE2 on vascular health, further translational and clinical studies are warranted to dissect its role as a druggable target to mitigate the effects of poor metabolic control on endothelial function.

## Data Availability

No new data were created or analyzed in this study. Data sharing is not applicable to this article.
